# Expert consensus on hyaluronic acid injections for knee osteoarthritis: a patient-centered approach

**DOI:** 10.1007/s40520-026-03366-8

**Published:** 2026-04-01

**Authors:** Nicholas Fuggle, François Rannou, Emmanuel Maheu, Alberto Migliore, Nasser Al-Daghri, Majed Alokail, Patrick Ammann, Francis Berenbaum, Angie Botto-van Bemden, Maria Luisa Brandi, Nansa Burlet, Roland Chapurlat, Cyrus Cooper, Elaine Dennison, Nicholas C Harvey, Gun-il Im, Andreas Kurth, Radmila Matijevic, Daniel Messina, Jordi Monfort Faure, Régis P. Radermecker, Ralf Schmidmaier, Stuart Silverman, Julien Wegrzyn, Leith Zakraoui, René Rizzoli, Jean-Yves Reginster, Olivier Bruyère, Ali Mobasheri

**Affiliations:** 1 MRC Lifecourse Epidemiology Centre, University of Southampton, Southampton, UK; 2 Faculté de santé, Université Paris Cité, UFR de Médecine, Paris, 75006 France; 3 Service de Rééducation et de Réadaptation de l’Appareil Locomoteur et des Pathologies du Rachis, AP-HP. Centre-Université Paris Cité, Hôpital Cochin, Paris, 75014 France; 4 Paris. INSERM UMR-S 1124, Paris, 75006 France; 5 Service de rhumatologie, Hôpital St Antoine, AP-HP, et cabinet médical, Paris, France; 6 Rheumatology Unit, San Pietro Fatebenefratelli Hospital, Rome, 00189 Italy; 7 Chair for Biomarkers of Chronic Diseases, Biochemistry Department, College of Science, King Saud University, Riyadh, Kingdom of Saudi Arabia; 8 Protein Research Chair, Biochemistry Department, College of Science, King Saud University, Riyadh, 11451 Kingdom of Saudi Arabia; 9 Division of Bone Diseases, Geneva University Hospitals and Faculty of Medicine, Geneva, Switzerland; 10 Sorbonne University, AP-HP Saint-Antoine Hospital, INSERM, Paris, France; 11 Musculoskeletal Research International, Key Biscayne, FL USA; 12 University Vita-Salute San Raffaele, Milan and FIRMO Foundation, Florence, Italy; 13 Division of Epidemiology, Public Health and Health Economics, University of Liège, Liège, Belgium; 14 INSERM UMR 1033, Université Claude Bernard-Lyon 1, Hôpital E Herriot, Lyon, France; 15 NIHR Southampton Biomedical Research Centre, University of Southampton, Southampton, UK; 16 NIHR Oxford Biomedical Research Centre, University of Oxford, Oxford, UK; 17 Department of Orthopedics, Dongguk University Ilsan Hospital, 32 Dongguk- Ro, Goyang Gyeonggi-Do , 10326 Goyang-si, Republic of Korea; 18 Orthopaedic Institute Dres. Baron & Colleagues and Johann Wolfgang Goethe University, Frankfurt, Germany; 19 Faculty of Medicine, University of Novi Sad, University Clinical Center of Vojvodina, Novi Sad, Serbia; 20 IRO Investigaciones Reumatologicas y Osteologicas SRL Collaborating Centre WHO, University of Buenos Aires, Buenos Aires, Argentina; 21 Servei de Reumatologia, Hospital del Mar, Universitat Pompeu Fabra, Barcelona, Spain; 22 Department of Diabetes, Nutrition and Metabolic Disorders, Clinical Pharmacology, CHU Liege, Liege University, Liege, Belgium; 23 Department of Medicine IV, LMU University Hospital, LMU Munich, Munich , Germany; 24 OMC Clinical Research Center and Cedars-Sinai Medical Center, 8641 Wilshire Blvd, Suite 301, Beverly Hills, CA 90211 USA; 25 Service universitaire d’orthopédie et de traumatologie, Hôpital orthopédique, Centre hospitalier universitaire vaudois et Université de Lausanne, Lausanne, Switzerland; 26 University of Tunis El Manar, Tunis, Tunisia; 27 Research Unit in Public Health, Epidemiology and Health Economics, University of Liège, Liège, Belgium; 28 Research Unit of Health Sciences and Technology, Faculty of Medicine, University of Oulu, P.O. Box 5000, Oulu, FI-90014 Finland; 29 Department of Personalized Medicine, State Research Institute Centre for Innovative Medicine, Santariskiu 5, LT-08406 Vilnius, Lithuania; 30 Department of Joint Surgery, First Affiliated Hospital of Sun Yat-Sen University, Guangzhou, China; 31 Department for Health Sciences, Medicine and Research, Center for Regenerative Medicine, University for Continuing Education Krems, Dr.-Karl-Dorrek-Strasse 30, 3500 Krems, Austria

**Keywords:** Osteoarthritis, Knee, Hyaluronic Acid, Injection, IAHA

## Abstract

Hyaluronic acid (HA) is a linear polysaccharide which occurs naturally as a constituent of synovial fluid. The HA concentration in the joint decreases inexorably during the progression of knee osteoarthritis (OA) and so, for nearly five decades, HA has been trialled and used in the treatment of knee OA. There are strong data from clinical trials, meta-analyses and umbrella reviews to support the use of intra-articular hyaluronic acid (IAHA) in the treatment of patients with knee OA, though not in patients with acute, active OA experiencing a flare. The majority of the literature suggests that IAHA has a positive safety profile despite a few meta-analyses suggesting an increased risk of serious adverse effects. Further qualitative analysis integrating patient preferences for multi-modal and/or non-surgical management is required in order to further explore these findings. IAHA has been combined with a number of additional agents, including mannitol, sorbitol, chondroitin sulphate, tranexamic acid, polynucleotides and hybrid IAHA formulations. These show variable performance beyond the baseline effect of their constituents. It is crucial to consider the patient’s preference when considering treatments for knee OA. Specific to IAHA, patients seek minimally invasive, lower-risk, and non-steroidal options with at least moderate efficacy and advantageous safety profiles.

## Keypoints

Hyaluronic acid is a naturally occurring constituent of synovial fluid and there are strong studies supporting its use in the treatment of osteoarthritis (outside the context of disease flares).

The majority of literature supports a highly advantageous safety profile for hyaluronic acid in terms of adverse effect profile.

Intra-articular hyaluronic acid (IAHA) can be injected in combination with other agents; including mannitol, sorbitol, chondroitin sulphate, tranexamic acid, polynucleotides and hybrid IAHA formulations, which show potential efficacy beyond their constituents alone.

## Introduction

Osteoarthritis (OA) is a major public health challenge. Knee OA alone affects approximately 595 million individuals worldwide (Global burden of disease). This represents a 132% increase since 1990 (Global burden of disease) and the burden is expected to rise [1], with a 75% increase by 2050 [2]. At a population level this places a significant financial burden on health systems [3] and budgets, with the social cost of OA estimated at between 0.25% and 0.5% of Gross Domestic Product [4].

This is not just a condition with epidemiological and health economic burden, having substantial, detrimental effects on patients with pain and loss of mobility leading to a limitation of activities and restriction of participation. Although there is no effective cure, treatments have been developed and trialled in order to manage symptoms and, in some cases, aiming to modify the disease process and guidelines exist to navigate a multimodal approach to therapy [5].

Intra-articular hyaluronic acid (IAHA) has a rich literature, with a recent review of studies of knee OA treatment demonstrating that IAHA has been studied in nearly four times as many participants compared to corticosteroids (40,862 treated with IAHA vs. 11,245 treated with corticosteroids [6]). It has been considered as a treatment for OA since 1971 [7] with early studies in the equine field demonstrating efficacy [8, 9] and human studies reporting in the 1980s [10, 11]. Since then it has been variably recommended in international and domestic guidelines [12–14], though with some variation in approach and the time is ripe to reconsider the place of IAHA in the treatment of knee OA, considering patient preferences for avoidance of steroids and surgery.

In January 2025, the European Society for the Clinical and Economic Aspects of Osteoporosis, Osteoarthritis, and Musculoskeletal Diseases (ESCEO) convened a working group consisting of patients, rheumatologists, orthopaedic surgeons, physicians in physical and rehabilitation medicine, epidemiologists and researchers. The purpose of this working group was to consider the latest qualitative and quantitative evidence regarding the use of IAHA in the treatment of knee OA.

This review article will set the scene by describing the epidemiological burden of OA (particularly of the knee), before highlighting the concept of ‘The Window of Opportunity’ for identifying patients in an early phase of their disease, when reversibility may still be possible. A summary of the current landscape of recommendations (and the proposed role of HA) will be followed by an exposition of the efficacy and safety of IAHA alone, and in combination with other agents. Finally, we will highlight the patient perspective of HA as an intra-articular therapy for knee OA.

### Window of opportunity

The concept of a ‘window of opportunity’ is associated with a desire to identify individuals in pre-disease, or in an early stage of disease, when non-surgical intervention has the ability to change the course of a condition and, potentially, even reverse a disease trajectory. This concept is well-established in some disease areas, with impaired glucose tolerance identified prior to the development of diabetes mellitus [15], with hypercholesterolaemia treated to improve cardiovascular risk [16], and with early inflammatory arthritis clinics aiming to identify and treat patients with inflammatory arthritis prior to the development of long-term, irreversible structural damage and joint erosion [17].

In OA, the window of opportunity has been mooted as the period after clinical symptoms have developed, but before irreversible structural changes are observed on radiographs [18]. The rationale behind this is that once radiographic changes are apparent, joint homeostasis is lost, structural changes have occurred in consort with biochemical and inflammatory alterations, such that the patient’s trajectory to joint replacement is irreversible.

The aim, therefore, is to identify patients in this window, to allow their trajectory to be changed, their future quality of life improved and their morbidity reduced. In order to achieve this, an accepted definition of the ‘early OA’ which constitutes this window is required and efforts to achieve this are ongoing [19].

### The guideline landscape

There are a large number of guidelines for the treatment of knee OA emanating from international and domestic societies. Within this body of recommendations (summarised in Table 1), there is variation in the extent to which IAHA is recommended for the treatment of knee OA and in some guidelines, there is a clear distinction between the primary and the secondary care settings.

**Table 1:** A summary of the international and national guidelines on the use of hyaluronic acid in the treatment of knee osteoarthritis

Guideline	Year	Recommendation regarding Hyaluronic Acid in Knee OA
European Alliance of Associations for Rheumatology (EULAR)	2003	Recommendation is made for IAHA in the context of symptomatic slow-acting drugs for osteoarthritis (SYSADOAs) saying they “have symptomatic effects and may modify structure”
American College of Rheumatology (ACR)	2019	“Intraarticular hyaluronic acid injections are conditionally recommended *against* in patients with knee…OA”
Veteran Affairs/ Dept of Defence	2020	“Weak recommendation for the use in knee OA”
Osteoarthritis Research Society International (OARSI)	2019	Level 2, conditional recommendation for patients with comorbidities or after failure of core treatments
European Society for Clinical and Economic Aspects of Osteoporosis, Osteoarthritis and Musculoskeletal Diseases (ESCEO)	2019	“The ESCEO working group affords a weak recommendation to the use of IAHA in patients who have contraindications to NSAIDs, or if the patient is still symptomatic despite the use of NSAIDs”
National Institute of Health and Care Excellence (NICE, UK)	2014	“Do not offer intra-articular hyaluronan injections for the management of osteoarthritis”
American Academy of Orthopaedic Surgeons (AAOS)	2022	Moderate recommendation not for routine use
Arthroscopy Association of Canada	2019	“Intra-articular injections of high-molecular-weight (HMW) IAHA provide improved pain relief and the restoration of function compared with placebo and can be considered in patients with mild to moderate knee OA. Strength of recommendation: Good – A”
Royal Australian College of General Practitioners (RACGP)	2015–2018	Recommends against IAHA injections in this primary care context, primarily due to cost (not covered by Medicare)
Société Française de Rhumatologie (SFR)	2020	Recommends IAHA independent of molecular weight or number of injections
Società Italiana di Reumatologia (SIR)	2019	“Intra-articular injection of hyaluronic acid of different molecular weights may give symptomatic benefit with low toxicity and could help to reduce the NSAID use”
Società Italiana di Ortopedia e Traumatologia (SIOT)	2023	Recommends IAHA in chronic disease cases, not for acute, active disease
Pan-American League of Associations for Rheumatology (PANLAR)	2016	“Intra-articular injection of hyaluronic acid of different molecular weights has proven to be beneficial in the treatment of knee OA”
Turkish League Against Rheumatism (TLAR)	2018	“Patients with moderate–severe symptoms, functional capacity of either normal or minimally limited and/or radiologic grade of 2–3 may be treated with NSAIDs in case of response to acetaminophen is absent or insufficient. These patients may be treated with intraarticular hyaluronic acid even though the evidence about its efficiency is uncertain.” Ia
Malaysian Delphi Consensus	2021	Recommends IAHA for advanced pharmacological therapy (following background treatment of SYSADOAs and topical NSAIDs with paracetamol if necessary) in “knee without effusion”
German Guidelines	2018	Recommends IAHA after topical and oral NSAIDs if inadequate response to therapy

In terms of international guidelines, these mostly conditionally recommend the use of IAHA according to the patient’s phenotype and according to the clinical context (primary care versus secondary care). The 2019 updated guidelines from ESCEO weakly recommend the use of IAHA in the context of the failure or contraindication of non-steroidal anti-inflammatory drugs (NSAIDs) and in a second step after primary care [12]. The 2019 OARSI guidelines conditionally recommend IAHA for patients with comorbidities or after the failure of ‘core’ treatments (including topical and oral NSAIDs) [14, 20], and both the EULAR 2003 [13] and PANLAR 2016 [21] recommendations maintain a positive recommendation for the use of IAHA for symptomatic therapy. In all these international guidelines, the use of IAHA is devoted to specialists involved in the care of musculoskeletal diseases including rheumatologists, orthopaedic surgeons, and physicians in physical and rehabilitation medicine.

The most regularly cited domestic guidelines for knee OA are from the US, with the ACR 2019 guidelines conditionally recommending against it and the Veteran’s Association 2020 guidelines weakly recommending for the use of IAHA in the treatment of knee OA [22, 23]. Australian General Practitioner guidelines do not support the use of intra-articular injections for OA in primary care due to the health economic implications to Medicare in the context of primary care [24, 25]. The UK National Institute of Health and Care Excellence (NICE) guidelines also recommend against the use of IAHA in primary care [26]. German recommendations advocate the use of IAHA as a third line pharmacological agent, after topical NSAIDs and oral NSAIDs [27]. French rheumatology guidelines [28] recommend that IAHA ‘should be proposed’ in cases of symptomatic knee OA with ‘no or minimal joint effusion’, a sentiment echoed in Malaysian guidelines recommending the use of IAHA as an ‘advanced pharmacological’ intervention for knee joints with ‘no effusion’ [29], demonstrating a similar approach in European and Asian populations. Italian rheumatology recommendations acknowledge the possible symptomatic benefits of IAHA ‘of different molecular weight’ and reduced use of NSAIDs are acknowledged [30], with Italian orthopaedic recommendations suggest ‘considering’ the use of IAHA for chronic knee OA ‘in the absence of active acute disease’ [31].

This summary serves to demonstrate that all the guidelines dedicated to musculoskeletal specialists recommend the use of IAHA for the treatment of knee OA, after trialling NSAIDs and with (at most) a minimal joint effusion to exclude acute flares. On the other hand, in the context of primary care, all the guidelines recommend against the use of IAHA [32, 33].

### Efficacy

#### Symptomatic efficacy

The effect of IAHA on clinical symptoms has been extensively studied through clinical trials and meta-analysis reporting variable results elicited from both the original studies and analyses based on secondary synthesis.

A 2025 umbrella review (systematic review of systematic reviews) examined 22 systematic review articles, rating 4 with high quality, 1 with moderate quality, 3 with low quality and 14 with critically low quality using the AMSTAR-2 (Assessing the Methodological Quality of Systematic Reviews-2) criteria. Significant beneficial effects of IAHA were noted in 20 of the 22 reviews, including all of the high and moderate quality reviews (k = 5). However, there was a clear discrepancy between the reported beneficial outcome data and the conclusions of the authors, which were negative in 3 of the high/moderate quality reviews. For example, the effect size of -0.37 SD, corresponding to a 0.9 cm change on a 10 cm Visual Analogue Scale (VAS), was pre-specified in one review. This review was based on a 2010 review [34] that drew on 4 previous papers in OA published between 1999 and 2004, with IAHA achieving this moderate reduction in pain (-0.37, 95% CI -0.46, -0.28), and yet the conclusion was that ‘viscosupplementation’ had no effect [35]. When examining the reasons for these negative conclusions, it seemed to be due, in the main, to post-hoc analyses which were performed after the main review analysis, restricted to large studies (with ≥ 100 participants in each arm) resulting in a less than clinically significant effect [35, 36]. Exclusion of small studies risks excluding well-performed and high-quality literature, simply on the basis of size which is not in keeping with the aims of systematic review and meta-analysis.

For trials of intra-articular interventions in general, there are additional issues which may affect the ability to demonstrate clinical efficacy. The common use of saline as a placebo in intra-articular injection studies does not fulfil the requirements of a placebo intervention due to substantial effects on hydrostatic forces within the joint and the dilution of inflammatory cytokines in synovial fluid [37]. Additionally, the effect of a placebo on an outcome measure such as pain should be viewed in light of the effect size of widely accepted treatments such as paracetamol and NSAIDs.

#### Structural efficacy

Injected HA is detectable in synovial fluid for up to 7 days after administration, and yet the symptomatic benefits are observed more than 6 months post-injection [38]. This suggests that the effects within the joint are not only rheological or anti-inflammatory but direct modifying effects on cartilage quality and chondrocyte metabolism. This symptomatic benefit may not be directly caused by effects on cartilage, as demonstrated in the example of sprifermin, which has positive effects on cartilage but no discernible effect on symptoms after 2 years [39]. Micro-indentation studies in a rat model have demonstrated substantial IAHA penetration into the cartilage which persists after joint washout with significant improvements in the Young’s modulus [40]. These demonstrated effects are physicochemical, rather than mediated via cellular modification (as there were no cells present in the cartilage model) and may help to explain why the effects of IAHA persist for months after injection and why there is potentially less benefit in those patients with no (or very little) cartilage. It may be that these physicochemical effects operate in consort with influences on chondrocyte phenotype, manifested via alterations in matrix quality [41].

#### Other joints

Of course, IAHA is used in other joints for the treatment of OA. The strongest evidence (beyond the knee literature) is for the use in base of thumb OA [42] (with recommendation for particularly small volumes of 0.4-0.7 ml and as few injections as possible), with emerging literature for usage at the hip [43–46] and shoulder [47, 48] and limited evidence at the ankle [49] and temporomandibular joint. In these other joints, the efficacy of IAHA often surpasses corticosteroid injection in medium term outcomes and it appears to be well tolerated. One particular nuance to consider when using IAHA in the hip (and other joints) is to ensure that injections are completed not less than 3–6 months prior to total hip replacement due to the risk of infection [50]. Beyond the knee, there is a general necessity for high-quality, large-scale RCTs with standardized protocols which will be essential to validate findings across all joint types.

#### Predicting response to hyaluronic acid

IAHA should not be considered a treatment by default, but as an active and forward step in the multimodal management of knee OA. The EUROVISCO group has performed important work in attempting to identify demographic and clinical factors which are predictive of response [32, 33].

Those factors associated with a potentially less pronounced response include older patients (over 70 years), obese individuals and those with more advanced (severe radiographic) OA. There may be less effect in those with particularly patellofemoral knee OA or those with effusions (as identified in the above guidelines) [32, 33].

Recent recommendations from EUROVISCO include recommendations for considering IAHA for the treatment of symptomatic knee OA regardless of age (including in the elderly > 90 years), for those with moderate to severe obesity (BMI ≤ 40), diabetes mellitus (type 1 and 2) and for those with concurrent history of gout (provided that it is not acutely active at the time of injection) [32, 33]. It can also be considered as a viable treatment for those with Kellgren–Lawrence (KL) grade 2–3 tibiofemoral knee OA, and those with symptomatic, early knee OA (with normal radiographs, in the ‘window of opportunity’ we allude to above). Patients with mild to moderate effusions (of 1–10 mL) can be considered but the group moderately recommends against the use of IAHA in knee OA flares.

#### Safety

There has been some controversy regarding the adverse event (AE) profile of IAHA and, in order to dissect this further, we will begin by defining some key terms and concepts.

AEs during clinical trials are defined by any untoward medical event which occurs in a patient receiving an investigational product, however, it is worth emphasising that this event does not necessarily have a causal relationship with the study treatment. Serious AEs (SAEs) are any AE which results in death, is life-threatening, requires hospitalisation (or prolonged existing hospitalisation), and/or results in persistent or significant disability or congenital abnormality or birth defect. Causality, in the case of an AE, is assessed by medically-qualified clinicians as part of a safety advisory board.

There are reviews of the literature which demonstrate an extremely beneficial safety profile of HA. An important network meta-analysis of 74 trials (*n* = 13,042), regarding 18 IAHA products in patients aged 45–75 years old confirmed systemic safety of IAHA with no major concern. The most frequently reported adverse event was transient, rapidly resolving, local pain or inflammation after injection with a prevalence of 8.5% [51]. A narrative review stated that IAHA ‘can result in minor transient local reactions… at a rate of 2–4%’ and ‘very few reports of severe side-effects’ [52]. A network meta-analysis found no significant difference in safety profile between IAHA, platelet-rich plasma, corticosteroid injection and placebo in terms of ‘treatment-related side events’ [53] and a meta-analysis of randomized trials found that ‘compared with IA saline, IAHA was associated with an increased risk of non-serious and transient local reactions’ [54]. A systematic review and meta-analysis draws attention to the quality of evidence in regard to safety and say that there can be ‘low to moderate certainty’ of a lack of ‘any safety issue’ [55] and a further review suggests that IAHA may reduce the use of NSAIDs and opioids [56] (with indirect benefits on safety).

However, some meta-analyses have suggested an association with SAEs. A meta-analysis of 14 trials (*n* = 3667 patients) reported an increased risk of SAEs (relative risk (RR) 1.41, 95% CI 1.02–1.97), though this was only based on 8 of the 14 trials [35]. It is worth noting that these ‘SAEs’ were later described as ‘most frequent disorders’ with 2 gastrointestinal events occurring in IAHA patients (compared to 8 events in controls), 5 cardiovascular systems events in IAHA patients (vs. 2 in controls), 6 cancer events (vs 0 in controls) and 4 musculoskeletal system events (vs 2 in controls) [35]. No hypothesis for causality is proposed. An update of this meta-analysis found similarly (SAE odds ratio (OR) 1.86, 95% CI 1.16 –3.03) [57].

A key element which is lacking from these meta-analyses is a qualitative approach to understanding the SAEs and a robust exposition of potential causative influence of IAHA. Unless clear causation is demonstrated, the current status quo should be accepted and the majority of the evidence points towards a beneficial safety profile of IAHA in the treatment of knee OA [58].

#### Combination therapy

There is an established and growing literature regarding the combining of IAHA with corticosteroids, polyols, chondroitin sulphate and other agents.

#### Corticosteroids

Both corticosteroids and IAHA have anti-inflammatory actions when administered intra-articularly and there is evidence from pre-clinical studies to support the assertion that combining these agents has an additive effect (beyond IAHA alone) in reducing gene expression of inflammatory cytokines (including matrix metalloproteinase-3 (MMP-3) and tumor necrosis factor alpha (TNF-α)) and increasing cell volume in osteoarthritic chondrocytes [59]. A meta-analysis of efficacy of corticosteroid-IAHA combined therapies (k = 8) demonstrated that the combination led to reductions in Western Ontario and McMaster Universities Osteoarthritis Index (WOMAC) pain scores at 2–4 weeks, 24–26 weeks and 52 weeks compared to IAHA alone [60]. No significant differences in safety profile were observed between the two interventions [60]. Additionally, no significance difference between the various corticosteroid-IAHA formulations was observed [60]. In terms of alterations in effect based on the type of steroid used, there are no direct comparative studies, however, triamcinolone hexacetonide and triamcinolone acetate are the corticosteroids associated with the least modification of rheological properties of hyaluronic acid [61].

#### Other combinations

In the 2017 ESCEO review of IAHA, it is stated that “some of the preparations include different concentrations of additives, such as mannitol, sorbitol, or chondroitin sulfate” [62]. The map of IAHA-combination therapies has extended substantially since then, with the addition of hybrid IAHA and some novel developments in polynucleotides and tranexamic acid.

Mannitol is a polyol (derived from mannose) reactive oxygen species (ROS) scavenger and, as such, has the potential to reduce degradation of injected HA leading to increased residence within the joint [63]. In vitro models, using hydrogen peroxide as a source of ROS, have demonstrated maintenance of elastic and storage moduli (measures of the rheological performance of a material) of an IAHA-mannitol combination over 30 min compared to deterioration of both parameters with IAHA alone [63]. A clinical trial of HANNOX-M (a mannitol-IAHA combination containing 31 mg of 1-1.5 MegaDalton (MDa) IAHA and 70 mg Mannitol in 2 mL) versus Bio-IAHA (20 mg of 2.3–3.6 MDa IAHA in 2 mL) demonstrated non-inferiority over 6 months (during which 3 injections were administered) in terms of change in WOMAC pain scores (Mean (SD) variation HANNOX-M = − 4.4 (3.8) mm, Bio-HA = − 4.5 (4.3) mm) [64]. Another randomized trial compared Ostenil Plus (2.0% IAHA (MW range 1 to 2000 kDa) + 0.5% mannitol and a 2.0 mL volume) with Synvisc One (0.8% of hylan G-F 20, avian crosslinked HA gel (80% hylan A + 20% hylan B) with a mean MW of 6 000 kDa and a 6 mL volume), which also demonstrated non-inferiority over 6 months of follow-up in a population of 292 knee OA patients [65].

Sorbitol is also a polyol, and diastereomer of mannitol. It is derived from glucose and is slightly more soluble than mannitol. The anti-inflammatory action of a sorbitol-IAHA combination therapy, SynolisTM V-A (IAHA 20 mg/mL (MW 2 MDa) with Sorbitol 40 mg/mL) has been demonstrated in vitro via the abrogation of IL-1β induced catabolism, inflammation and ROS production via significantly discernible effects on Nitric Oxide, inducible nitric oxide synthase (iNOS), prostaglandin E₂ (PGE_2_) and MMP-13 in a human chondrocyte model of OA, suggesting benefits of the formulation, including restoration of the redox status [66]. Non-inferiority of a single injection of Synolis VA (80 mg IAHA (MW 2MDa) with 160 mg Sorbitol) compared to Synvisc-One (48 mg Hylan GF-20) has been demonstrated via a clinical trial for knee OA (*n* = 202) with reductions in WOMAC pain scores (mean ± SD: Synolis VA -29.2 ± 24.1, Synvisc-One − 31.6 ± 25.5, *p* = 0.57) and an approximately equal proportion of responders in each group (Synolis VA 79%, Synvisc-One 77%) [67]. A sub-group analysis (the sorbitol-IAHA arm only, *n* = 91) of this trial demonstrated that baseline functional status was a predictor of response to the sorbitol-IAHA combination [68].

Chondroitin sulphate is an important constituent of the cartilage, supporting hydration and elasticity of the tissue [69]. Supplementation manifests an anti-catabolic function on the cartilage matrix via inhibition of the NF-κB with downstream reduction in pro-inflammatory cytokine secretion [69]. The rationale for combining chondroitin to IAHA is not simply to achieve an additive effect but also so that it can increase viscosity (by increasing the molecular size via hydrogen bond formation), leading to increased tissue adherence and improved rheological properties [69]. In a rat model of OA (achieved via ACL ligament transection) chondroitin-IAHA combination was more effective than IAHA alone or chondroitin sulphate alone in reducing inflammatory cytokine secretion (TNF-α, interleukin-6 (IL-6), and interleukin-1 beta (IL-1β)) and producing higher levels of collagen-II and aggrecan [69]. Open-label, single-arm studies have demonstrated clinical efficacy of chondroitin-IAHA combination in reducing WOMAC pain over 6 months (*n* = 112) using 3 injections of Arthrum^®^ (40 mg chondroitin, 40 mg IAHA) [70], in reducing pain intensity and severity (*n* = 21, 6 months) using 1 injection of Hialurom Hondro^®^ (90 mg chondroitin, 60 mg IAHA) [71] and reducing VAS pain score, WOMAC pain, stiffness and physical function over 6 months (*n* = 83) using SINOGEL^®^ (48 mg chondroitin, 72 mg IAHA) [72].

Low and high molecular weight IAHA may have differing actions within the joint which may be complementary, leading to the development of hybrid molecular weight IAHA combinations. High molecular weight IAHA is potentially viscosupplementary within the joint (though the concept of viscosupplementation is debated due to the persisting symptomatic benefit of IAHA long after it is cleared from the joint), whilst low molecular weight IAHA may help to improve the physical ease of injection, and facilitate greater penetration into the synovial extracellular matrix (ECM) and act as biosupplementation (via chondroprotection and stimulation of endogenous IAHA synthesis) [73]. An extensive systematic review synthesised the available studies of hybrid molecular weight IAHA finding rapid reduction of pain from a single injection over 24 weeks of follow-up in 692 participants with radiographic knee OA [74], significantly superior pain and clinical outcomes compared to PRP in a cohort of end-of-career professional footballs with degenerative cartilage lesions [75] and greater efficacy for pain reduction for hybrid molecular weight IAHA compared to high molecular weight IAHA in a group of 48 obese patients [76].

Highly purified polynucleotides (extracted from trout gonads) have provoked interest as potential donors of nitrogen base precursors of nucleotides and nucleosides to allow development of chondrocytes and mesenchymal stem cells. This has led to the production of polynucleotide-IAHA combination therapy which has demonstrated significantly better efficacy in reducing WOMAC pain scores compared to IAHA alone at 2, 6 and 12 months (*p* < 0.01), but not at the 24-month follow-up (*p* = 0.09) [77].

Tranexamic acid is a lysine analogue, inhibitor of plasminogen activator and inhibitor of MMP-mediated proteolysis. These properties have led to a product combining tranexamic acid with IAHA for the treatment of iodoacetate-induced OA in rats [78]. This combination demonstrated significant benefits symptomatically (for paw withdrawal threshold) and structurally (for OARSI scores) compared to IAHA alone.

In conclusion, there is convincing evidence of efficacy of polyol-IAHA combination therapy and, to a lesser extent, with hybrid molecular weight IAHA combination therapy (which would benefit from further head-to-head comparisons with non-hybrid formulations). The evidence base for chondroitin sulphate-IAHA combinations in blinded, randomised, multi-arm studies and the use of polynucleotide-IAHA and tranexamic acid-IAHA combinations is an interesting area of development for future research.

#### Patient Preferences for Intra-Articular Hyaluronic Acid (IAHA) Treatment

Clinicians should aim not only to deliver evidence-based care but to empower patients to become informed participants in their treatment decisions [79]. In the context of IAHA, this requires both a clear explanation of the risks and benefits, and an understanding of the diverse formulations available. Particularly distinctions to be drawn include molecular weight and cross-linking, which may influence efficacy and tolerability. Patient education should be provided in a stepwise manner to ensure comprehension without cognitive overload, especially when patients are being asked to choose between different IAHA options (high vs. low molecular weight formulations).

Importantly, incorporating patient preferences into shared decision-making is not only ethically aligned with person-centered care but is also supported by health economic and regulatory frameworks. The International Society for Pharmacoeconomics and Outcomes Research (ISPOR) emphasizes the importance of aligning clinical interventions with what patients value in their care experience [80, 81], including comfort, accessibility, and functional outcomes [80]. Empathy, active listening, and the elicitation of individual goals and expectations are essential when planning intra-articular therapy [82].

Across diverse populations, patients consistently report that safety is a top priority. IAHA products are generally well tolerated, and patients prefer treatments with a well-established safety profile and low risks of inflammation or infection [14]. Rapid symptom relief is also highly valued, especially when OA symptoms interfere with mobility, independence, or quality of life [83]. Although IAHA may take longer to act than corticosteroids, some patients report symptom improvement within one to two weeks, and those who understand the expected time course are more satisfied with outcomes [82].

The duration of benefit is also important. Patients favour interventions that offer sustained relief, ideally for up to six months, reducing the frequency of clinic visits and procedural discomfort [84]. Studies have shown that patients are willing to pay more for injections [32] that provide longer-lasting efficacy [81]. Importantly, convenience plays a pivotal role. Patients typically prefer single-injection formulations over multi-injection regimens to minimise time commitment, discomfort, and the anxiety associated with repeated clinic visits [81].

Technical aspects of injection also affect patient comfort. Larger injection volumes may prolong the procedure and contribute to pain, while larger-gauge needles are often associated with more discomfort. To optimise the patient experience, clinicians should consider using 22- or 25-gauge needles and low-volume formulations, particularly for small joints such as the fingers or the trapeziometacarpal joint.

Financial considerations are also critical. IAHA injections are variably reimbursed across health systems, and for patients paying out of pocket, cost-effectiveness becomes an essential factor. Many patients are willing to accept out-of-pocket expenses if they perceive meaningful improvements in pain, function, or quality of life [68, 85]. Transparent discussions about cost, value, and expected outcomes are imperative for fostering trust and ensuring satisfaction.

## Conclusions

In conclusion, efficacy and safety data support the use of IAHA in the treatment of knee OA in secondary care and the inclusion of IAHA in international and national guidelines. Overall findings thus far suggest that the optimal benefit to risk ratio is in those with early OA, who have the most to gain from reduced pain and improved performance to maintain muscle strength and joint function. There is burgeoning research examining the combining of IAHA with additional agents and further studies in this area will assist with distilling out the particular patient phenotypes that are likely to benefit best from particular formulation. The preferences of patients with knee OA are of paramount importance and should be prioritised when using shared decision-making to develop treatment plans.

## Data Availability

No datasets were generated or analysed during the current study.
